# A retrospective study of the prevalence and characteristics of dens invaginatus in a sample of the Turkish population

**DOI:** 10.4317/medoral.18285

**Published:** 2012-12-10

**Authors:** Kaan Gündüz, Peruze Çelenk, Emin M. Canger, Zeynep Zengin, Pınar Sümer

**Affiliations:** 1 Associate Professor, Department of Dentomaxillofacial Radiology, Ondokuz Mayıs University, Faculty of Dentistry, Samsun, Turkey; 2 Professor, Department of Dentomaxillofacial Radiology, Ondokuz Mayıs University, Faculty of Dentistry, Samsun, Turkey; 3 ….

## Abstract

Objective: The purpose of this study was to assess the prevalence of dens invaginatus and to classify the types of dens invaginatus in a sample of the Turkish population.. Study Design: A retrospective study was performed using periapical and panoramic radiographs of 5355 patients who presented to the Department of Oral Diagnosis and Radiology at the Ondokuz Mayıs University Dentistry Faculty between January 2009 and December 2010. Maxillary and mandibular anterior teeth were evaluated for the presence and characteristics of dens invaginatus. Statistical evaluation of the presence of dens invaginatus related to gender was performed by the Pearson chi-squared test. Results: Dens invaginatus was observed in 116 of 4556 subjects, with a frequency of 2.5%. There was only one periapical lesion in teeth with type I dens invaginatus, but 8.1% of patients with type II and 87.5% of patients with type III dens invaginatus had apical periodontitis at the time of referral. There were 116 (72%) females and 32 (27%) males with dens invaginatus. Conclusion: This data represents the only study carried out in a large population in Turkey, and no dens invaginatus was found in mandibular teeth. The most commonly observed type of dens invaginatus was type I (69.8%).

** Key words:**Dens invaginatus, dens in dente, dental anomaly, Turkish.

## Introduction

Dens invaginatus, or dens in dente, is a tooth abnormality first described by a dentist named Socrates in 1856 (1). It is also re-ferred to as invaginated odontome, dilated gestant odontome, dilated composite odontome, and dentoid in dente (2). Dens invaginatus is an enamel-lined developmental malformation that occurs as a result of an invagination of the dental papilla during the soft tissue stage of tooth development (3,4). Any of the teeth in the maxillary and mandibular arch may be affected by dens invaginatus, but the maxillary lateral incisors are most commonly affected (5-7). This abnormality has a frequency of 0.04% to 10% in the general population. This variation is probably due to geographical differences and different diagnostic criteria and methods of investigation (8).

Different classifications have been suggested to describe dens invaginatus. The most common classification system was first proposed by Oehlers (2), who classified dens invaginatus into three categories according to the depth of penetration and communication with periapical tissues or the periodontal ligament. Type I is an enamel-lined minor invagination occurring within the coronal part of the crown without an extension beyond the cemento-enamel junction. Type II is an enamel-lined invagination extending into the root beyond the cemento-enamel junction and remains as a blind sac. Type III is an invagination penetrating through the root to form an additional apical or lateral foramen.

There are a limited number of studies reporting the prevalence of dens invaginatus in the literature (7). Therefore, the purpose of this study was to assess the prevalence of dens invaginatus and to classify the types of dens invaginatus in a sample of the Turkish population. This information provides the dental practitioner with information about the types of teeth that are more likely to exhibit technical difficulties associated with endodontic treatment.

## Material and Methods

A retrospective study was performed using periapical and panoramic radiographs of 5355 patients who presented to the Department of Oral Diagnosis and Radiology at the Ondokuz Mayıs University Dentistry Faculty between January 2009 and December 2010. Orodental, medical (syndromes and systemic diseases) and demographic characteristics of patients were obtained in a standardized manner from clinical records.

Patients with incomplete records or poor quality panoramic and periapical radiographs were excluded.

A final sample of 4556 patients was selected (2536 males, 2020 females). Patient ages ranged from 13 to 65 years, with a mean age of 22.4 years. Presence of syndromes and systemic diseases were noted. Variations in the crown shape and other dental anomalies were also recorded by radiographs and dental records showing abnormal crowns in teeth with dens invaginatus. The relationship between dens invaginatus and sex was also investigated.

This study was based on retrospectively evaluation of radiographs. Thus, no ethical approval was obtained from the local ethical committee since only the achieve data were used for the study. However, before taking any radiograph or intra/extra-oral examination, patients gave their informed consent prior to radiography and examinations according to the principles of the Helsinki Declaration, including all amendments and revisions.

Maxillary and mandibular anterior teeth were evaluated with periapical and panoramic radiographs to determine the presence and classification of dens invaginatus using Oehlers classification (2) who classified dens invaginatus into three groups according to the depth of the penetration and communication with periapical tissue or periodontal ligament: Type I is an enamel-lined minor invagination occurring within the coronal part of the crown without an extension beyond the cemento-enamel junction. Type II is an enamel-lined invagination extending into the root beyond the cemento-enamel junction and remains as a blind sac. Type III is an invagination penetrating through the root to form an additional apical or lateral foramen.

**Figure 1:**
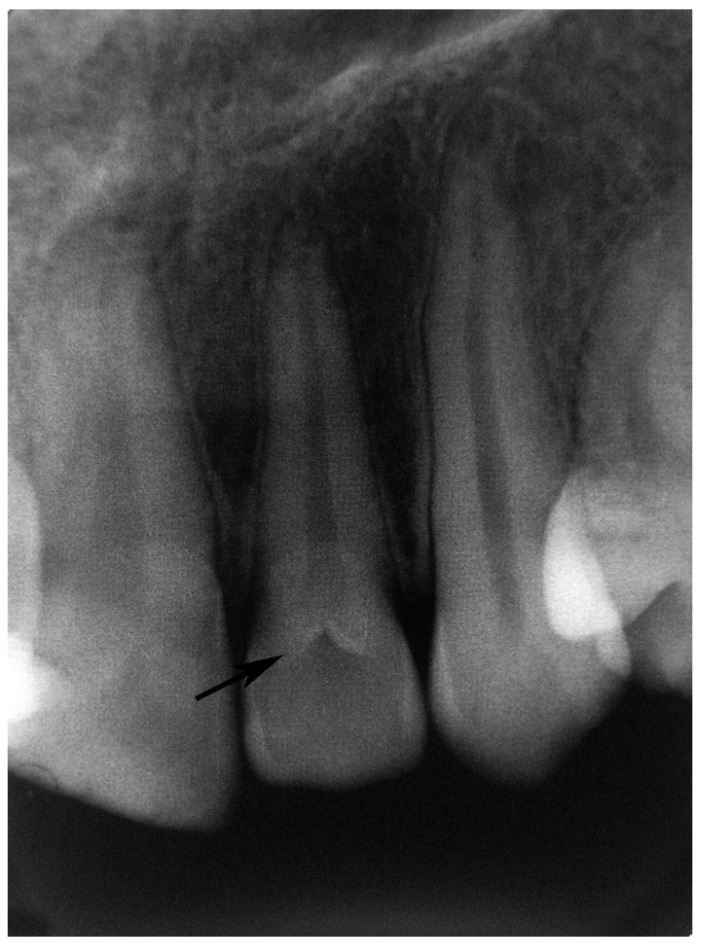
Periapical radiography shows a type I dens invaginatus according to the classification of Oehlers (arrow shows dens invaginatus).

**Figure 2:**
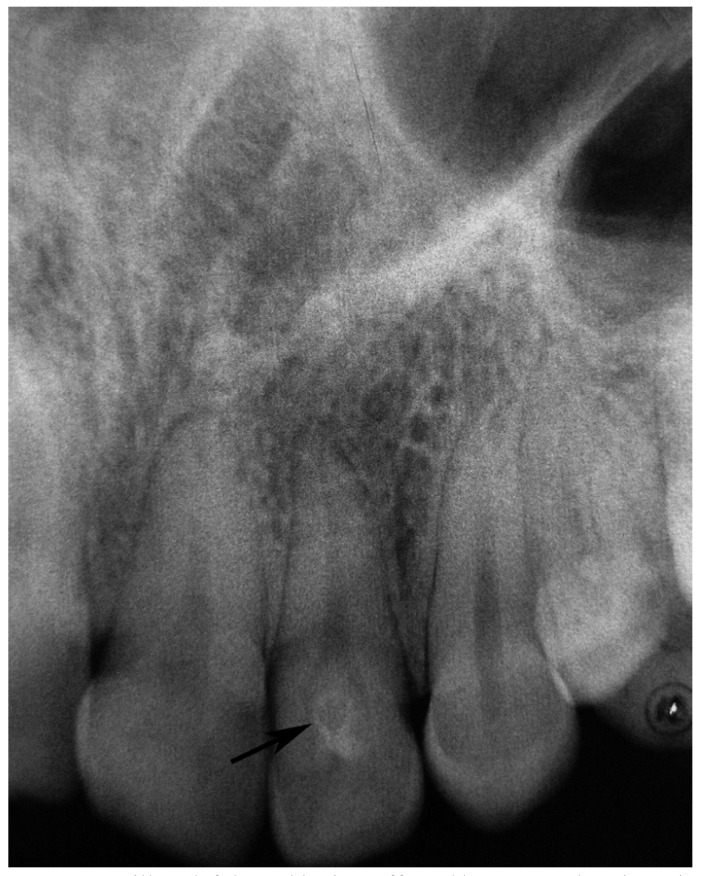
Maxillary left lateral incisor affected by Type II dens invaginatus (arrow).

**Figure 3:**
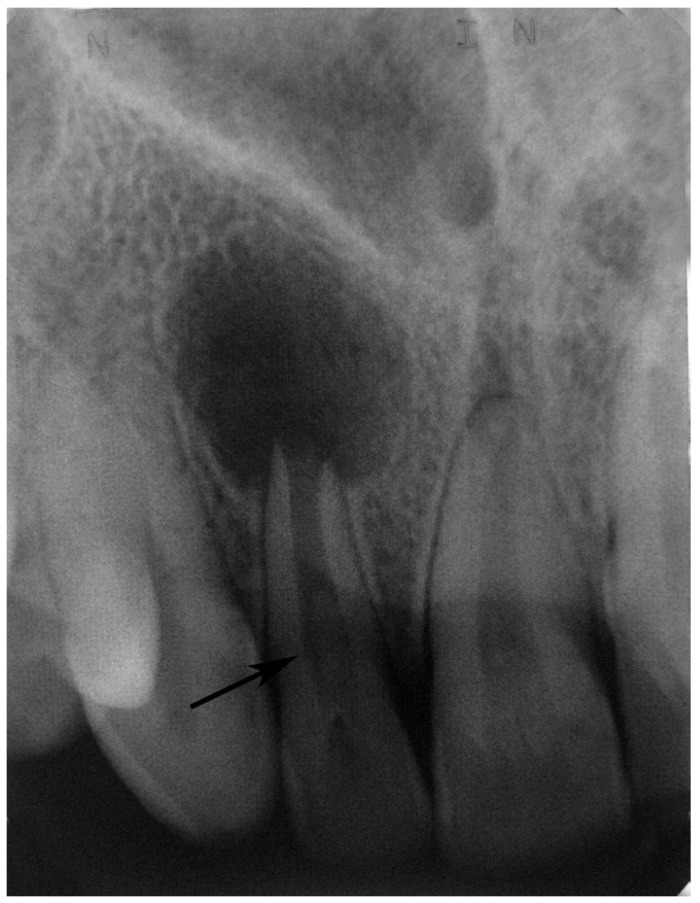
Maxillary right lateral incisor with periapical lesion affected by Type III dens invaginatus (arrow).

Radiographic examples of the different types of dens invaginatus are shown in (Figs. 1,2,3). Irregular widening of the periodontal ligament space and the presence of periapical pathosis in invaginated teeth were assessed using the ‘Periapical Index’ (9) based on radiographs taken at the time of referral to the clinic.

All radiographs were reviewed in a dark room with an x-ray viewer (Illuminator 5000; RP Beard Ltd, London, UK) and evaluated independently by two examiners. A separate assessment of the radiographs was performed by each examiner, and the types of dens invaginatus were agreed upon according to the suggestions of the more experienced investigator. A final evaluation was performed, and a collective decision was made to determine whether or not the tooth had dens invaginatus and the type.

Statistical evaluation of dens invaginatus as it related to gender was carried out using the chi-square test.

## Results

Dens invaginatus was observed in 116 of 4556 subjects, with a frequency of 2.5. Of the patients screened, 2020 (44%) were female and 2536 (56%) were male. There were 116 (72%) females and 32 (27%) males with dens invaginatus ( Table 1). Dens invaginatus was detected bilaterally in 77 patients and unilaterally in 39 patients.

**Table 1:**
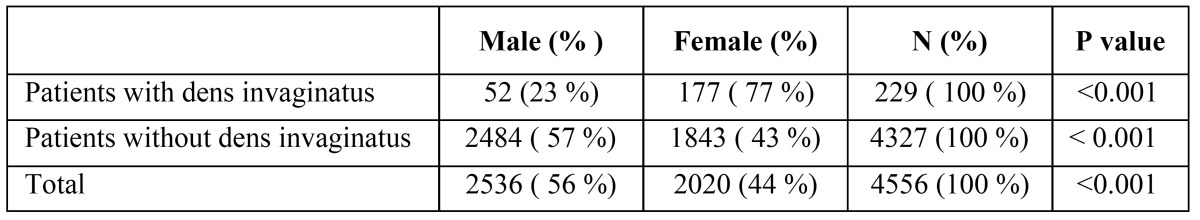
Distribution of patients with dens invaginatus.

The distribution of teeth with dens invaginatus is presented in  Table 2. There were 22126 maxillary and 21941 mandibular teeth examined for the presence of dens invaginatus. The anomaly was detected primarily in maxillary lateral incisors (184 out of 7388), followed by maxillary central incisors (44 out of 7726) and maxillary canines (1 out of 7012). No dens invaginatus was detected in mandibular teeth.

**Table 2:**
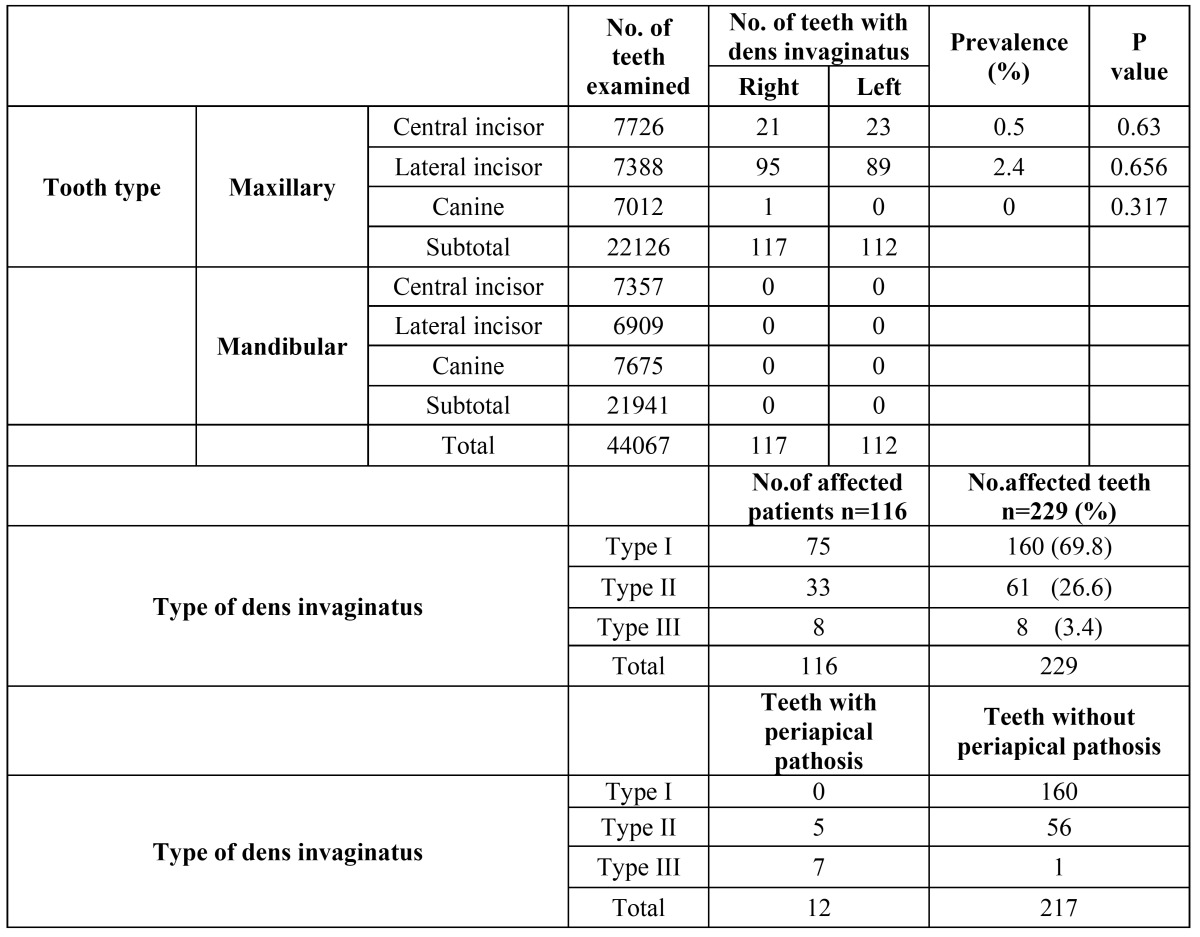
The distribution of the teeth with dens invaginatus.

The distribution of the type of dens invaginatus is shown in  Table 2. The most commonly observed dens invaginatus was type I (69.8%), followed by type II (26.6%) and type III (3.4%).

The number of teeth with and without periapical pathosis and the type of dens invaginatus is presented in  Table 2. There was only one periapical lesion in teeth with type I dens invaginatus, but 8.1% of patients with type II and 87.5% of patients with type III dens invaginatus had apical periodontitis at the time of referral.

Other dental abnormalities that were seen in patients with dens invaginatus are also recorded. Dens invaginatus was mostly encountered with hypodontia and taurodontism. Associations with other systemic diseases and syndromes were not detected.

## Discussion

Although the etiology of DE is still uncertain, it appears that both genetic and environmental components exist. Rushton (3) suggested that the cause was embryological, stemming from the stimulation and subsequent proliferation and ingrowth of cells of the enamel organ into the dental papilla during development. In contrast, some authors considered that the problem was the result of the retardation of a focal group of cells, with surrounding cells continuing to proliferate normally (10). Oehlers (2) suggested that the problem was the result of external forces exerting an effect on the tooth germ during development. Such forces could be from adjacent tooth germs, for example, the central incisor or canine, which develop at least six months prior to the lateral incisor (10). Other external factors such as trauma (3) and infection (1) have also been suggested as potential causes.

During tooth development, tooth morphogenesis is affected by the ectomesenchymal signaling systems that occur between the dental papilla and the internal enamel epithelium (11). These signals have specific roles, such as the regulation of growth and the folding of the enamel organ (12). The absence of certain molecules can result in abnormally shaped teeth as well as in defects in the developing tooth germ (13). For this reason, the proposal that genetic factors may be the cause of dens invaginatus has some credibility (14). Support for this possible cause also comes from one reported case of an individual lacking chromosome 7q32 who presented with dens invaginatus in addition to other dental abnormalities such as hypodontia (15). There is further support from a clinical study of 3,020 Swedish children that reported 2.7% of patients with dens invaginatus. Of the subjects in this study with the condition, 43% of their parents and 32% of siblings also had evidence of the abnormality (16). Additional evidence suggesting a genetic influence comes from the fact that the invaginations appear to have a limited variation (2) and can occur in a number of teeth in the same individual (7) or in siblings (4). There also appears to be an increased incidence of the condition in mongoloid groups and a lower incidence in negroid groups (2), and clustering of other genetically determined dental anomalies has been observed (7,10).

Dens invaginatus malformations are usually detected accidentally. A patient will generally not discover this anomaly unless clinical signs appear, such as an acute dentoalveolar abscess or sinus tract. Most cases of dens invaginatus are discovered radiographically (1,7,8,17-19). In this retrospective study, the clinical characteristics of dens invaginatus were determined using clinical records and radiographs. Because all of the patients did not have complete periapical radiographs for the maxillary and mandibular posterior teeth and the panoramic radiographs did not give a clear picture of posterior teeth with dens invaginatus, only maxillary and mandibular anterior teeth were evaluated. Therefore, the results of this study do not represent a complete assessment of the mouth.

The reported prevalence of dens invaginatus is between 0.3% and 10%, and it represents a problem in 0.25% to 26.1% of the subjects examined (7-11). The wide variation in reported prevalence may be explained by the different cohorts studied, geographical differences and different diagnostic criteria and methods of investigation (10). In this study, dens invaginatus was observed in 2.54% of patients evaluated, which is consistent with the results of several studies carried out in Turkey and other countries. Cakici et al. (17) found a prevalence of 1.3% out of 1184 patients, Ulmansky and Hermel (19) and Hamasha et al. (20) reported a prevalence of 2% and 2.95%, respectively. However, a study by Kirzioglu and Ceyhan (18) on 2477 patients found the prevalence of dens invaginatus to be 12%. The authors suggested that the high prevalence ratio might be related to regional, communal and genetic factors.

The appearance of the symmetric dens invaginatus was considered to be a common finding by some authors. Bilateral appearance is not unusual and occurs in 43% of all cases (1). Bilateral occurrence was reported to be in conjunction with several dental anomalies, including taurodontism, microdontia, gemination, and dentinogenesis imperfect (18,20). In this study, 51.7% of dens invaginatus cases were bilateral and occurred with additional dental anomalies in some teeth. Maxillary anterior teeth, particularly lateral incisors with a deep foramen, must be carefully examined for dens invaginatus, even in the absence of clinical symptoms. Due to the frequent bilateral occurrence of dens invaginatus, teeth should be bilaterally examined.

Dens invaginatus is seen on maxillary lateral incisors, maxillary central incisors, and maxillary canine teeth. Dens invaginatus in the mandible is extremely rare (1,20). The results of this study are comparable with previous reports (1,17,18,20-23), with the maxillary lateral incisors (80.3%) as the most frequently affected anterior teeth, followed by the maxillary central incisors (19.2%) and the maxillary canines (0.4%). This data represents the only study carried out in a large population in Turkey, and no dens invaginatus was found in mandibular teeth. However, there are a few case reports in the literature of dens invaginatus in premolar and molar teeth in the mandible.

Oehlers’ classification system is the most popular for dens invaginatus. This system is based on a two-dimensional radiographic image and may underestimate the true extent and complexity of the invagination (11). Using Oehlers’ classification, the prevalence of the different types of dens invaginatus was reported as follows: type I, 79%; type II, 15%; and type III, 5%. Alani and Bishop (16) also reported the prevalence of type I to be 79%. The results of our study are similar to those of previous studies, with type I as the most prevalent at 69.8%. The coronal and root parts of a tooth may be sites where dens invaginatus occurs (18). Dens invaginatus is more prevalent at the coronal part of the tooth, and type I, type II, and type III proportions were estimated to be 94%, 3% and 3%, respectively. At the time of referral, 87.5% of the patients with type III dens invaginatus and 8.1% of the patients with type II dens invaginatus had periapical pathosis. Oehlers’ classification is applied using radiograms, which are two-dimensional images of an object. There are limitations associated with patient positioning and angle of the x-ray, which prevents visualization of the true extent and complexity of an invagination. A tooth that radiographically reflected as a type II invagination might have been histologically categorized as a type III.

Dens invaginatus may occur with various dental anomalies, particularly hypodontia and dens evaginatus (18). Additionally, association with supernumerary teeth is rare; only few cases have been found in the literature (24-28). In this study, no dens invaginatus was found in supernumerary teeth. Concomitant presence of dental anomalies in a single patient indicates common etiological factors (24). It has been suggested that anomalies, such as dens invaginatus/evaginatus, fusion, and gemination, are the result of hyper or hypo activity of the dental lamina and occur more frequently in the anterior region of the jaw (25,26). Our study indicated hypodontia as the most frequent anomaly co-occurring with dens invaginatus.
